# Spurious Drugs

**Published:** 1848-02

**Authors:** 


### EDITORIAL DEPARTMENT.

*Spurious Drugs.*—We are gratified to perceive that attention is being directed to the adulteration of medicines, and the means of protecting the profession and the public against the impositions which are so largely and extensively practiced. It is ascertained that the importation of spurious drugs has been, and still is carried on in a systematic manner, and to an enormous extent. The Editor of the New-York Journal of Medicine, who has evinced a zeal in the investigation of this subject which entitles him to the warmest thanks of the public and profession, in an article which was copied in a late number of this journal, exposes several instances of this species of fraud; and in a communication in the last number of the New-York Journal by a wholesale dealer in drugs in the city of New-York, the fact is fully admitted, without any conscientious compunctions, the communication, in fact, being intended to exculpate the said firm from blame, on the ground, forsooth, that they keep genuine drugs as well as spurious, leaving the purchaser to determine which are genuine and which are spurious by the prices affixed to each! Our contemporary very clearly exposes the speciousness of casuistry which would transfer the moral responsibility of the traffic from the wholesale to the retail dealer in these cases. Admitting that the retailer, and the dispenser of medicinal preparations, are fully aware that the articles they sell and use are spurious, does this render the wholesale dealer any the less a party to the nefarious business? Is the criminality of the latter diminished by the fact that others are equally criminal? But as our friend of the New-York Journal remarks, it is by no means probable that the matter is fully understood by all who purchase of the importer, and of the apothecary. In a large proportion of cases, undoubtedly, the apothecary, and the country physician, suppose that the articles purchased are genuine articles. It is impossible, in the nature of things, that the price affixed should be a reliable criterion. If this were sufficient, how easy to sell spurious articles at as high price as genuine! The dealer who can reconcile his conscience to engage at all in the traffic of adulterated medicines, would seldom find it difficult to place as high price upon them as upon those which are pure, when his gains would thereby be quadrupled, or more. The subterfuge offered in the communication to which we allude, is too shallow and pitiful to deserve serious refutation. It could only spring from that moral obliquity which the admitted fact of dealing in spurious drugs involves. But apothecaries and practitioners are by no means blameless in this matter. If they are not voluntary parties to the fraud, they are not sufficiently careful and discriminating. It is too much the custom to buy articles which are the most cheaply furnished, and to patronize establishments which sell the cheapest, without due examination of the purity of medicinal preparations. We do not deny that both apothecaries and practitioners are, in many instances, justly chargeable with participating in deliberate impositions. The crime is not limited to the importer; but it is fair to presume, that the practitioner very seldom prefers spurious medicines to genuine, for, if he has no better motive, his own interests are involved to an extent vastly beyond the amount of money saved by the cheaper rate at which they are purchased. The attention of the Profession being now in various ways directed to this subject, it is to be hoped the evils will be, in some measure, at least, removed. The first, and most important point, is for physicians who dispense medicines to bestow more care upon the examination and testing of those which they purchase, and to patronize only honest and competent druggists. Practitioners who send prescriptions must ascertain that they are carried to apothecaries of known integrity and skill. But inasmuch as the utmost vigilance will not prove entirely protective, it is an important point to prevent the importation of spurious drugs. We can see no way in which this can be effected but by legally appointed inspectors of medicines. In one of the early numbers of this journal we proposed and advocated this measure, and commented on the inconsistency of requiring an inspection of pork, flour, and tobacco, and, at the same time, making no provisions against frauds in the sale of adulterated drugs, the latter being certainly not less injurious than inferior tobacco, and assuredly less easy to be detected by the purchaser and consumer. This plan is now generally recommended.

The *Philadelphia College of Pharmacy* has taken up the subject, and issued a Circular, and form of petition to Congress, copies of which are herewith appended. We commend the subject to the Profession, and hope that it will receive general co-operation. Petitions might also, as it seems to us, with propriety be circulated among the public generally, for their interests, after all, are more deeply involved in the matter than those of the medical practitioner. The subject is one of so much importance that if presented to the attention of Congress we cannot imagine that it will fail to be properly appreciated by that honorable body.

The following are the circular and form of petition referred to:—

### CIRCULAR.

The undersigned, a committee of the Philadelphia College of Pharmacy, respectfully ask your attention to the annexed resolution and memorial to Congress, as passed and issued by the body they represent, and request, if the proposed object meets your approbation, that you will co-operate with them to the extent of your ability in effecting the reformal aimed at, and which are but too necessary.

The College, whilst fully impressed with the importance of the movement commenced by the New York College of Pharmacy, as set forth in their published circular, believe that we are laboring under very serious evils of domestic origin, over which Congress have no control. To meet these difficulties as far as possible, the College, in addition to memorializing the National Legislature, have instructed this committee to prepare a paper for general circulation, on the adulterations with which the drug-market is at present infected, suggesting with each the best known methods of identification, having a view to the greatest simplicity of means, in order that their application may be conducted, by even unskilful hands, as far as possible.

This portion of the labors of the Committee wiil be much facilitated, as well as rendered more widely useful, if those who have connection directly or indirectly with the drug-market will communicate any knowledge they may possess relative to the subject, or send specimens of adulterated drugs or chemicals that may come under their notice, to the Chairman, or any member of the Com mittee.

We would therefore again solicit your co-operation in furtherance of the proposed reforms and are very respectfully yours,

DANIEL B. SMITH,
CHARLES ELLIS,
AMBROSE SMITH,
THOMAAS P, JAMES,
EDWARD PARRISH,
ROBERT BRIDGES,
JOSEPH CARSON,
WILLIAM PROCTER, JR.

*Philadelphia*, 11*th month* (*Nov.*,) 1847.

At a Special Meeting of the Philadelphia College of Pharmacy, held Eleventh month 1st, 1847, it was

Resolved, That copies of the Memorial passed this evening be transmitted by the Committee to the various Colleges of Medicine and Pharmacy of this country, with an urgent request that they will co-operate in obtaining the proposed results.

*To the Honorable Senate and House of Representatives.*

The memorial of the Philadelphia College of Pharmacy, respectfully represents:

That one of the chief objects of the establishment of their institution, was to "direct attention to the quality of drugs brought into the market," with a view to correct the evils arising from the introduction and sale of spurious and sophisticated articles.

That they have, from time to time, by the proper education of young men in their school of Pharmacy, by exposing frauds of various kinds when discovered, and by the publication of a *Journal*, which assumes a high tone in its exposition of these abuses, done much to correct the evils spoken of.

That it has now become notorious amongst druggists, that of late, important drugs and medicines are especially adulterated in foreign countries, for sale in this country, and pass daily through the Custom house, to be disseminated by ignorant or unprincipled dealers, to the great detriment of our citizens.

That whilst we are aware there are domestic abuses of grave character to correct, and whilst it is the ceaseless exercise of this and similar institutions to do away with, by elevating the character of the practitioners of Pharmacy, and disseminating a knowledge of these abuses, and the means of detecting them—a work which has more especially engaged us at this time,—we believe that Congress possesses the power, in the enactment of a suitable law, to exert a highly beneficial influence on the character of imported drugs, by rendering it impossible to introduce them without undergoing inspection by a proper and qualified inspector, whose duty it should be to ascertain the real character of the drugs and chemicals destined for medical use, and keep such records, that honest dealers may ascertain at any time the character of the importations and take means of protection against imposition. We believe that such a law would, within a moderate period, exhibit a very evident influence on the quality of importations; as it is generally only necessary that such sophistications be known, to be condemned by the majority of dealers.

Your memoralists therefore ask of your honorable bodies, that a law be enacted, embracing the appointment of a proper inspector at each chief port of entry, whose duty it shall be to examine all the importations of drugs, medicines, and chemicals used as medicines, and, if necessary, have them properly tested, keep a full record of such inspections, including the names of the parties, which shall be open for consultation to the druggists, apothecaries, physicians, and others, concerned; or adopt such other measures as in your wisdom may seem best adapted to prevent the evil complained of.

*President.*

} *Vice Presidents.*

*Secretary.*

